# Automated Adaptive Approach for Specular Highlight Removal in Digital Dentistry: A Proof-of-Concept Study for Preserving Tooth Surface Texture

**DOI:** 10.3390/jcm15135319

**Published:** 2026-07-07

**Authors:** Ji Su Han, Sung-Ae Son, Il-Ho Park, Eun-Ha Jung, Jeong-woo Lee, Seok-Woo Park, Jae-Seung Jeong

**Affiliations:** 1 Department of AI Convergence, Sahmyook University, Seoul 01795, Republic of Korea; lllinous@syuin.ac.kr; 2 Department of Conservative Dentistry, Dental and Life Science Institute, School of Dentistry, Dental Research Institute, Pusan National University, Yangsan 50612, Republic of Korea; sungae@pusan.ac.kr; 3 College of Pharmacy, Sahmyook University, Seoul 01795, Republic of Korea; parkilho@syu.ac.kr; 4 Department of Dental Hygiene, Kyungpook National University, 2559 Gyeongsang-daero, Sangju 37224, Republic of Korea; jeunha725@knu.ac.kr; 5 Department of Dental Services Management and Informatics, Seoul National University, Seoul 03080, Republic of Korea; dentmast@naver.com; 6 Department of Preventive Dentistry and Public Oral Health, Oral Science Research Institute, Yonsei University College of Dentistry, Seoul 03722, Republic of Korea; 7 R&D Center, Vatech, 13, Samsung 1-ro 2-gil, Hwaseong-si 18449, Republic of Korea

**Keywords:** specular highlight removal, digital dentistry, intraoral photography, image inpainting, image preprocessing, dynamic threshold adjustment, tooth surface texture

## Abstract

**Background/Objectives:** Digital intraoral photography is widely used for clinical documentation, longitudinal monitoring, and AI-assisted dental image analysis. However, specular highlights caused by saliva and intense illumination can obscure tooth texture and compromise image fidelity. This study aimed to develop an automated method for removing specular highlights from intraoral images while preserving tooth surface texture. **Methods:** A three-stage pipeline consisting of adaptive threshold prediction, mask generation, and image inpainting was proposed. Initially, the Hue, Saturation, Value (HSV) statistical features were extracted from each image and used to train a regression model that predicts an image-specific threshold. Subsequently, the predicted threshold was applied in the CIE LAB color space, followed by a condition-based dynamic adjustment algorithm to refine the mask area and distribution. Finally, an Aggregated Contextual Transformation (AOT)-based generator network was used to restore the masked regions. **Results:** The proposed dynamic adjustment reduced over-masking compared with regression-only processing and better preserved tooth surface texture. Pixel distribution analysis demonstrated a lower distributional discrepancy, with the Wasserstein distance reduced from 2.9601 to 1.3505 and the Kullback–Leibler divergence reduced from 0.3451 to 0.0618. In the clinical expert evaluation, the proposed method was preferred in 69.5% of the 200 evaluation responses, and the preference difference was statistically significant (*p* < 0.001). **Conclusions:** As a proof-of-concept study conducted under controlled conditions using synthetic images, the proposed pipeline reduced specular highlights while better preserving tooth surface texture than the baseline approaches. These findings suggest that the pipeline may support standardized preprocessing of dental image datasets, although broader applications such as long-term monitoring and AI-assisted diagnostic workflows require validation on real clinical photographs.

## 1. Introduction

In contemporary dentistry, digital intraoral imaging, including clinical photography, plays an important role in objective clinical documentation, patient monitoring, and supporting clinical decision-making [1]. Notably, digital imaging enables the documentation of subtle enamel-surface irregularities or morphological variations that may be difficult to assess consistently by unaided visual inspection alone [2]. This capability provides reliable clinical evidence for quantitatively comparing and analyzing changes in the tooth structure and conducting long-term follow-up observations. Consequently, it substantially aids clinicians in presenting visual evidence to patients based on more objective records, thereby enhancing the precision of treatment. However, in clinical practice, the presence of saliva in the oral cavity, complex curvatures of the tooth surface, and intense illumination during imaging often result in strong specular highlights. Because these conditions are largely unavoidable during intraoral photography, specular highlights are not an isolated occurrence but a problem that recurs across routine clinical imaging. These highlights act as critical noise, degrading the quality of clinical records by causing the loss of intrinsic color and texture information of the teeth and inducing structural distortions [3,4,5]. When such reflections obscure the color and texture of the enamel, subtle surface changes and early carious lesions may be misinterpreted, and the reliability of photographic comparison during long-term follow-up is reduced. Moreover, when images containing specular highlights are used directly for training or inference in AI models, the reflected regions can act as artificial high-intensity patterns that are unrelated to the true color and texture of the tooth, functioning as noise that may be confused with the genuine signal the model is intended to learn. Conventional intensity-based thresholding approaches typically attempt to remove specular highlights using fixed thresholds. However, these methods are limited because they do not adequately account for the variability in the imaging angle and illumination intensity [5,6]. Employing a single criterion poses a significant risk of either retaining specular highlights or excessively losing normal dental tissue information, thereby distorting the tooth surface texture. Furthermore, the manual designation of masking areas in clinical settings is time-consuming and costly, rendering it unsuitable for large-scale data standardization purposes [7,8]. Therefore, to optimize the efficiency of digital dentistry, there is an urgent need for automated technology that selectively removes only specular highlights and restores lost areas to match the intrinsic texture of teeth.

Efforts to detect and remove specular highlights have a substantial history, yet they have not focused on dental images. Among classical image processing methods, techniques have been developed for the real-time detection of specular reflections based on statistical color space features [9], and a survey has summarized the broader progress of these detection and removal techniques [5]. In the medical imaging domain, an early approach segmented specular highlight regions in endoscopic images and restored them through inpainting [4]. Deep learning-based approaches, in contrast, have developed mainly around natural images, including multitask networks that jointly learn the detection and removal of specular highlights [3] and methods that leverage large-scale synthetic data to improve restoration quality [10]. More recently, this trend has extended to medical imaging, with a two-stage hierarchical network proposed to detect and remove specular highlights on tissue and instrument surfaces in endoscopic and surgical images [6]. These methods, however, have largely targeted natural images or non-dental medical images such as endoscopic and surgical images, and specular highlight removal in dental intraoral images, with an emphasis on preserving the fine surface texture of enamel, has not yet been actively investigated.

Accordingly, this study proposes a novel algorithm that integrates automatic threshold prediction and dynamic mask adjustment algorithms to address the issue of specular highlights in intraoral images without requiring user intervention or manual adjustment.

First, it helps preserve the fine surface texture of the teeth during specular highlight removal. Selectively removing only the specular highlights while maintaining the original surface texture is a key factor in keeping clinical images representative of the actual oral condition. If the fine curvatures of the tooth surface or the characteristic texture of the enamel are lost owing to over-masking during this process, the resulting image no longer faithfully reflects the patient’s oral condition. By reducing such distortions, this approach can provide a more consistent visual basis for comparing the same site over time, for example, when assessing the progression of lesions or tooth wear.

Second, the proposed method enhances the acquisition of advanced AI training data by eliminating manual bias and standardizing the preprocessing procedures. Traditional approaches, which involve manually designating masking areas, often yield inconsistent results owing to the operator’s proficiency and subjectivity, thereby compromising the reliability of data archiving [7,8]. In contrast, the proposed pipeline mitigates user bias and supports more consistent data processing by automatically handling all images according to uniform, statistical criteria. Additionally, this approach offers a practical solution to the data bottleneck, which is a significant limitation in the development of next-generation dental diagnostic AI solutions. Although deep learning-based diagnostic models are rapidly advancing, they encounter substantial challenges in acquiring high-fidelity, large-scale datasets necessary for training [8,11]. By employing the automated and standardized preprocessing of this algorithm, the clinical resources expended on manual labor can be reduced, thereby maximizing the efficiency of data collection for AI training.

Taken together, the proposed approach contributes to digital dentistry by preserving fine surface texture during specular highlight removal and by reducing the manual bias inherent in conventional preprocessing, thereby supporting more consistent and reproducible dental image data. Therefore, this study aimed to develop and evaluate an automated adaptive framework for specular highlight removal in intraoral images.

## 2. Materials and Methods

### 2.1. Dataset

To ensure precise evaluation and consistent model training of the specular highlight removal algorithm, this study employed synthetic intraoral image data sourced from the AI Hub. The AI Hub pool comprised 145,140 high-quality synthetic intraoral images categorized by angle, which reflected actual patient scenarios and a variety of cases. This entire pool was not used in the present study. From this pool, 14,514 images were used for development of the HSV regression model, of which 452 images were manually labeled and served as the ground truth for training the model, while the remaining 14,062 images formed an unlabeled target set on which the trained model predicted thresholds, in order to confirm that it produced reasonable predictions across a broader distribution beyond the labeled data. These 14,062 images were used only at this threshold prediction step and were not carried through to mask generation, inpainting, or any of the final result metrics. A separate set of 2463 images, with no overlap with the 14,514 images, was used to apply and evaluate the three-stage pipeline. From these, 50 images were randomly drawn for the clinical expert evaluation. The 452, 14,062, and 2463 image sets were each drawn from distinct source cases, with no case appearing across multiple subsets, so that no data leakage occurred between the labeling and training stages and the evaluation stage. The composition of these subsets is summarized in Table 1. Synthetic data were meticulously constructed to facilitate the training and evaluation of the model by simulating the diverse dental complexities and illumination conditions encountered in real clinical settings [10,12].

**Figure 1:**
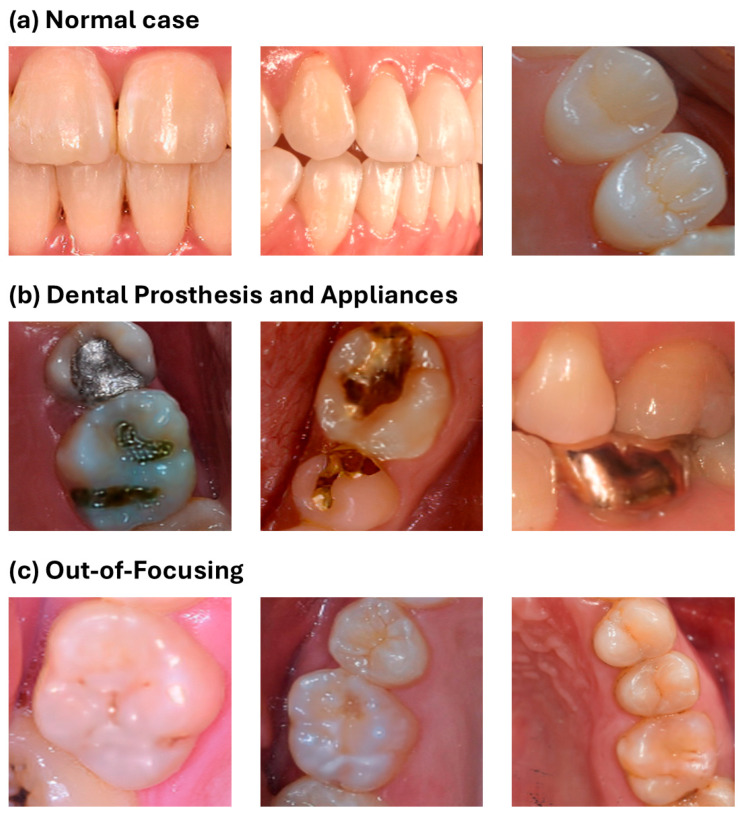
**Composition of the dataset and case classification used in the experiment**. The dataset is categorized into three types to ensure accurate evaluation of the algorithm. (**a**) Normal case showing natural tooth surfaces with clear focus, which is the primary target for the proposed method. (**b**) Dental prosthesis and appliances case containing artificial structures such as metal crowns and wires. (**c**) Out-of-focusing case where tooth contours and highlight boundaries are blurred. Only normal cases were utilized for training and evaluation to avoid structural distortions found in other categories.

To ensure the model’s versatility and prevent bias toward specific perspectives during the construction of the training dataset, images were selected in equal proportions from each case to uniformly represent images from each angle (FRONT, LEFT, RIGHT, UPPER, LOWER). Specifically, to concentrate on the specular highlights, which constitute the core objective of this study, and to minimize noise within the images, this study processed and utilized cropped images that specifically targeted areas where specular highlights were prominently visible rather than employing the entire image. The collected images were categorized into three groups based on their qualitative state and distribution characteristics of the specular highlights. Figure 1 illustrates the classification criteria for the entire dataset.

The “Normal” category pertains to standard dental photographs devoid of abnormalities, characterized by images where the surface comprises natural teeth with a clear focus. In contrast, the “Dental Prosthesis and Appliances” category encompasses images featuring artificial structures such as metal prostheses, resins, and orthodontic appliances, where the characteristics of specular highlights differ from those of the natural teeth. The “Out-of-Focusing” category includes images with a blurred focus, rendering the contours of the teeth and the boundaries of specular highlights indistinct. Notably, the “Dental Prosthesis and Appliances” and “Out-of-Focusing” categories exhibit characteristics where the distribution and properties of specular highlights are abnormally distorted or where boundary recognition is challenging, potentially impairing the specular highlight detection performance. Metallic and artificial materials produce reflection and saturation patterns that differ physically from those of natural enamel. Whereas enamel is a translucent structure with fine surface texture, metal prostheses and orthodontic wires present uniform and highly reflective surfaces, resulting in specular highlight intensity and distribution patterns that are fundamentally different. In strongly saturated regions, the underlying surface information itself is lost, making it difficult to distinguish areas degraded by specular reflection from the inherent brightness of the material. Applying the same restoration strategy to both natural tooth surfaces and prosthetic or appliance regions therefore risks altering the boundary or structure of the artificial material in a manner that deviates from reality. Given that the objective of this study was to optimize specular highlight removal on normal tooth surfaces, these two categories were excluded from the analysis and training processes, and only images from the “Normal” category were used for the training and evaluation of the proposed algorithm. Specular highlight removal for artificial structures such as prostheses and orthodontic appliances requires material-specific detection and restoration strategies and is regarded as an independent topic for future work.

### 2.2. Theoretical Background

#### 2.2.1. HSV Color Space

The Hue, Saturation, Value (HSV) color space offers a representation of colors that aligns more closely with human visual perception than the RGB (Red, Green, Blue) color space [13]. Specifically, Hue (H) denotes the type of color and is typically depicted as a circular structure ranging from 0° to 360°. Saturation (S) indicates the intensity of the color, representing the transition from achromatic to pure color. The Value (V) conveys brightness information, determining the lightness and darkness of the color. The HSV color space is advantageous because of its ability to independently separate brightness, color, and saturation, thereby facilitating the detection of specular highlight regions characterized by high brightness and low saturation [9,13]. In this study, HSV statistical information was used as the input for the regression model to predict the optimal threshold for each image, enabling the detection of specular highlights.

#### 2.2.2. CIE LAB Color Space

**Figure 2:**
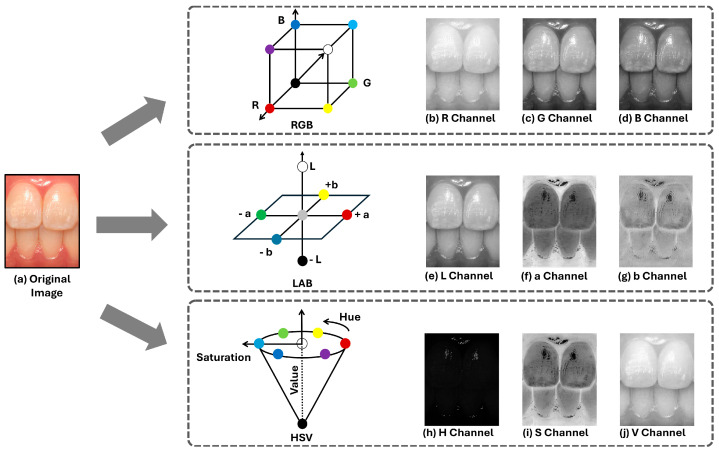
**Conceptual diagram of HSV and LAB color space conversion.** Structural differences between the RGB, LAB, and HSV color spaces and the decomposed channels used in the proposed pipeline. (**b**–**d**) The separated red, green, and blue channels of the original RGB image (**a**), showing that color and brightness information are correlated. (**e**–**g**) The channels of the image converted from (**a**) to the LAB color space, where the L channel is utilized for mask generation. (**h**–**j**) The Hue, Saturation, and Value channels of the image converted from (**a**) to the HSV color space, where HSV statistics are used for optimal threshold prediction.

The $L^{*}$, $a^{*}$, $b^{*}$ (LAB) color space, established by the International Commission on Illumination (CIE) in 1976 [14], is a standard designed to ensure perceptual uniformity by numerically modeling human visual perception [13]. The $L^{*}$ channel independently represents lightness, which reflects the human visual bias. Based on the opponent color theory, the two chromaticity channels, $a^{*}$ and $b^{*}$, correspond to the green–red axis and the blue–yellow axis, respectively. A significant advantage of the LAB color space is its capacity to express brightness and color information independently [13,15]. In contrast, in RGB or HSV color spaces, masking operations are limited because of the mixing of brightness and color, which is influenced by the illumination environment. However, the LAB space is particularly suitable for analyzing illumination intensity based solely on variations in the $L^{*}$ channel. Utilizing these characteristics, this study generated a binary mask that accurately delineated specular highlight regions by applying the optimal threshold predicted by the regression model to the $L^{*}$ channel. Figure 2 provides a visual comparison of the structural differences between the RGB, HSV, and LAB color spaces.

### 2.3. Overview of the Proposed Pipeline

**Figure 3:**
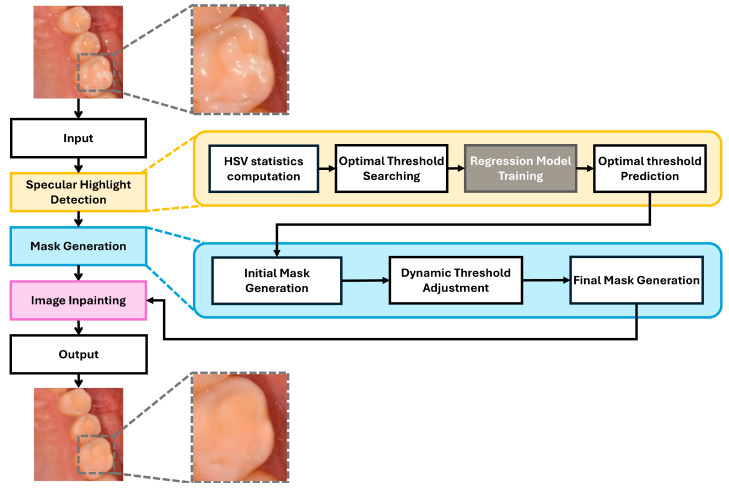
**Overview of the proposed specular highlight removal pipeline.** Schematic of the three-stage automated process: Specular Highlight Detection (HSV-based threshold prediction), Mask Generation (initial mask with dynamic threshold adjustment), and Image Inpainting (AOT-GAN restoration of the masked regions).

This study presents a three-stage pipeline developed to autonomously and precisely remove specular highlights from dental images, thereby eliminating the need for user intervention. This process consists of three interconnected stages: specular highlight detection, mask generation, and image inpainting, as illustrated in Figure 3.

**Figure 4:**
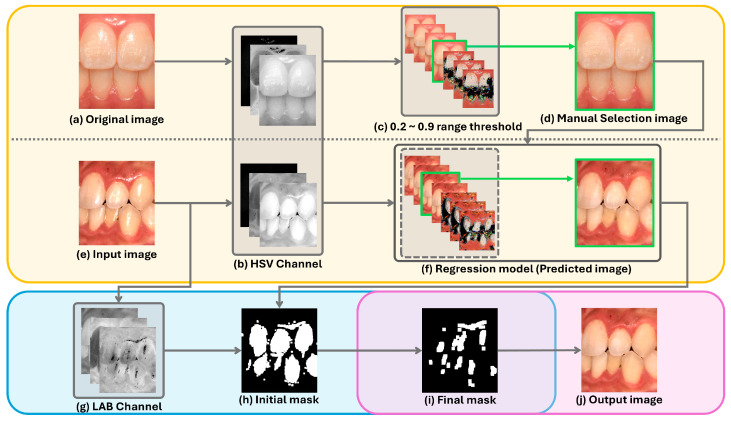
**Framework of the proposed specular removal approach.** End-to-end illustration of the process, where the yellow, blue, and purple boxes correspond to the “Specular Highlight Detection,” “Mask Generation,” and “Image Inpainting” stages defined in Figure 3, respectively. (**a**) Original input image. (**b**) Split HSV channels of (**a**) for statistical analysis. (**c**) Removal results for thresholds from 0.2 to 0.9. (**d**) Manually selected optimal-threshold result used as the training label. (**e**) Input image for threshold prediction and mask generation. (**f**) Removal result using the regression-predicted threshold, which informs the initial mask. (**g**) Split LAB channels of (**e**). (**h**) Initial binary mask from the predicted threshold. (**i**) Final binary mask after dynamic threshold adjustment. (**j**) Final output inpainted with the AOT-GAN model.

The implementation results of the logical flow of the algorithm on actual image data are shown in Figure 4. Figure 3 delineates the control flow of the pipeline, and Figure 4 presents empirical evidence of the data transformation process at each stage, thereby demonstrating the effectiveness of the system. Notably, Figure 4 visualizes the principal steps outlined in Figure 3 by distinguishing them with yellow, blue, and purple boxes, thus illustrating the functional connectivity between the two diagrams.

The initial phase of the algorithm, termed “specular highlight detection,” commences with the acquisition of statistical data by transforming the original image Figure 4a into the HSV color space, as shown in Figure 4b. Subsequently, to facilitate the training of the regression model, candidate images were produced by applying various thresholds ranging from 0.2 to 0.9 based on the aforementioned data, as illustrated in Figure 4c. The resultant image Figure 4d, in which the specular highlights are most effectively removed according to the clinical evaluation, is selected and employed as the training data. The model trained in this manner autonomously predicts the optimal threshold for a new image Figure 4e to generate an output similar to Figure 4f. This procedure, denoted by the yellow box, constitutes the fundamental mechanism of “automation” that enables standardized preprocessing without the need for expert intervention.

The subsequent “mask generation” phase involves converting the input image Figure 4e into the LAB color space, as depicted in Figure 4g, followed by the creation of an initial mask Figure 4h by applying the threshold predicted by the regression model from the preceding stage. Thereafter, a final mask Figure 4i, which preserves the tooth surface texture, is computed by implementing a “dynamic threshold adjustment” algorithm. This algorithm precisely calibrates the threshold by analyzing the area ratio and distribution of the mask, as indicated by the blue box, thereby supporting data consistency by flexibly adapting to diverse clinical environments.

In the “image inpainting” phase, represented by the purple box, the AOT-based Generator Network [16] uses the confirmed final mask. The model meticulously aligns the mask area with the texture of the adjacent dental tissue to produce the final output image Figure 4j. This process facilitates the acquisition of high-fidelity data by naturally reconstructing the detailed surface texture obscured by specular highlights. As this three-stage pipeline is designed to account for the varying intensity and distribution of specular highlights in each image, it aims to provide consistent and stable performance without requiring the user to specify the areas or manually adjust the thresholds. Consequently, this technology contributes to the establishment of a standardized preprocessing procedure in clinical settings while enhancing the convenience and efficiency of the entire process.

### 2.4. Specular Highlight Detection

The initial phase of this study involved the automatic detection of specular highlights in dental images. These highlights predominantly arise due to the curvature of the tooth surface and intense illumination, typically exhibiting low saturation and high brightness [5,9]. To accurately capture these characteristics, the input image was initially transformed from the RGB color space to the HSV color space. The HSV color space is particularly advantageous for effectively distinguishing bright and low-saturation specular highlight regions, as the Value and Saturation components can be managed independently in response to changes in illumination [9,13]. In contrast, the RGB color space combines color and brightness information, complicating the clear differentiation of specular highlights from the general structures. Consequently, the HSV color space was employed in the specular highlight detection phase of this study.

**Figure 5:**
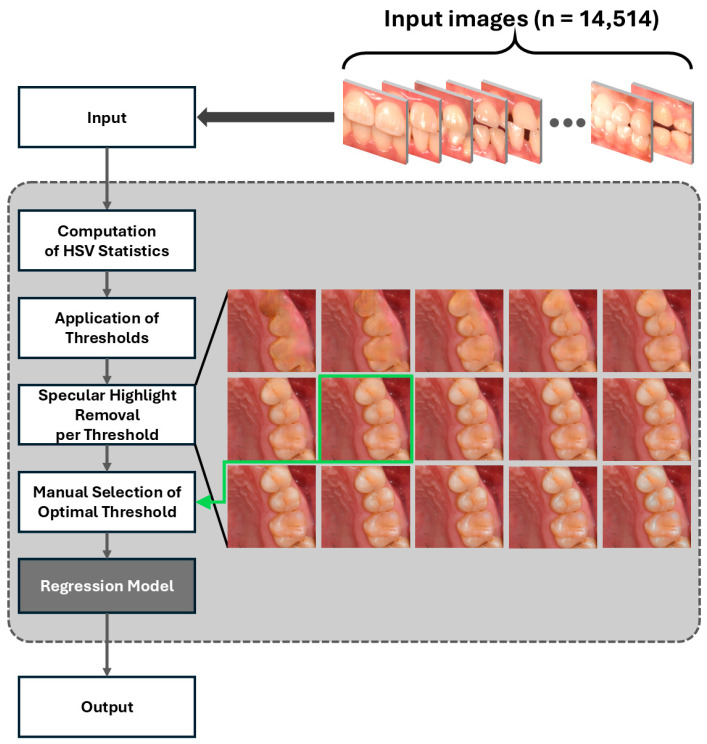
**Regression model training pipeline.** Workflow for establishing the ground truth and training the threshold prediction model. Input images are converted to the HSV color space and statistical features (min, max, mean, std) are computed. Candidate results are generated across thresholds from 0.20 to 0.90, and the optimal threshold is selected by visual inspection. The HSV statistics and corresponding optimal thresholds form the dataset used to train the regression model for automatic prediction.

Following the conversion to the HSV color space, statistical metrics, namely the minimum, maximum, mean, median, and standard deviation, were computed for the H, S, and V channels of each image. This produced 15 statistical features per image, comprising 5 statistics for each of the 3 channels, and these 15 features were used as the input to the regression model without any additional feature selection or dimensionality reduction. Unlike traditional methods that employ fixed thresholds [4,5], this study generated masks by applying various thresholds at 0.05 intervals, ranging from 0.20 to 0.90. For each of the 452 ground truth images, the optimal threshold was selected as the value that most effectively removed the specular highlights while preserving the surrounding tooth surface texture. This selection was performed independently by five of the authors, four of whom have clinical experience in dental settings that informed their visual judgments, while the remaining author contributed from a technical image analysis perspective. Calibration and consensus procedures among the raters, as well as formal inter-rater reliability metrics, were not applied in the present study, and this is noted as a limitation in the Discussion. By applying this process across the 452 ground truth images, a labeled dataset comprising the HSV statistical features and the corresponding optimal threshold for each image was constructed, and a regression model was trained on this dataset. The search process for determining the optimal threshold is shown in Figure 5.

A Linear Regression model was used for threshold prediction. Because this model has no hyperparameters such as a learning rate, a number of trees, or an early stopping criterion, no such tuning was applicable. To evaluate the model, the 452 labeled images were divided into training and test sets at an 8:2 ratio, and 5-fold cross-validation was additionally performed on the training set to verify the generalization performance of the model. All image processing was conducted at the original image resolution, without resizing or down-sampling. The entire pipeline was implemented in Python version 3.9.21. OpenCV version 4.13.0 was used for image processing and color space conversion between the HSV and LAB spaces, scikit-learn version 1.1.3 was used to train the Linear Regression model for threshold prediction, and PyTorch version 2.8.0+cpu was used for AOT-based image inpainting. 

The model trained in this manner utilizes HSV statistical values as inputs for a new image to automatically predict the optimal threshold for effectively removing specular highlights from the corresponding image. This regression-based threshold prediction model is instrumental in enhancing the efficiency and reproducibility of the entire pipeline. Given that the intensity and distribution of specular highlights vary significantly based on the illumination conditions, tooth surface curvature, and imaging angles for each image, it is challenging to consistently eliminate specular highlights from all images using a single fixed threshold [5,10]. This algorithm addresses the issue of variance between images by automatically predicting the optimal threshold for each image based on HSV statistical information, thereby providing stable specular highlight detection performance even in diverse everyday environments.

Specular highlight detection serves as a fundamental preliminary processing stage that influences the input quality of subsequent stages, namely, mask generation and image inpainting. The accuracy and stability of this stage are crucial for the overall performance of the pipeline. The method proposed in this study not only mitigates the need for manual adjustments by automating the threshold-setting process but also significantly enhances the quality of the mask and inpainting outcomes by facilitating the consistent detection of specular highlights across all images.

### 2.5. Mask Generation

In the second stage, mask generation is executed through a sequence comprising initial mask generation, static adjustment based on the mask area, condition-based dynamic adjustment, and final mask generation, culminating in the creation of the mask employed in the subsequent inpainting stage. Initially, a binary mask that identifies the specular highlight region is generated by applying the optimal threshold predicted during the specular highlight detection stage. In this process, the LAB color space is utilized instead of the RGB or HSV color spaces to more accurately leverage the brightness information. The L channel of the LAB color space is particularly suitable for reliably detecting extremely bright areas, such as specular highlights, and generating binary masks because it represents lightness separately from chromatic components [13,14,15]. Consequently, this study isolates the specular highlight regions by focusing on the L channel of the LAB color space during the mask generation stage. Following the LAB conversion, the L channel is extracted, and the average brightness of the image is calculated. Subsequently, a binary mask detecting the specular highlight region is generated according to the following formula:

(1) $$Mask\left(x,y\right)=\{\begin{array}{c} 255\;\;\;\;\;\;\;\;\;\;\;\;if\;L\left(x,y\right)>\mu_{L}\times \left(1+T\right)\\ \;\;\;0\;\;\;\;\;\;\;\;\;\;\;\;\;\;\;\;\;\;otherwise\;\;\;\;\;\;\;\;\;\;\;\;\;\;\;\;\;\;\;\;\;\;\;\;\;\;\;\;\;\;\; \end{array}$$

In this context, $\mu_{L}\;$ represents the mean brightness value of the image, and T  denotes the optimal threshold determined by the regression model. This method for establishing the threshold is introduced because the predicted threshold typically falls within the range of 0.2 to 0.9, which implies that merely adding this value may not adequately capture the specular highlights. Consequently, regions exhibiting brightness greater than 1+T times the average brightness are identified as specular highlights. This approach involves initially securing a broad masking range, which is subsequently refined through meticulous adjustment.

**Figure 6:**
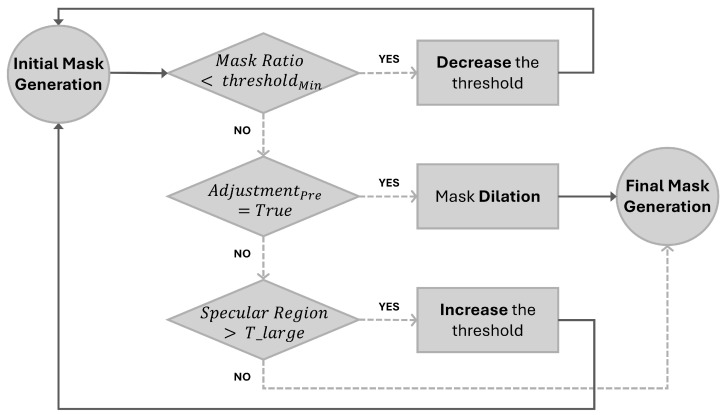
**Flowchart of the dynamic threshold adjustment algorithm.** The decision logic for refining the initial mask generated by the regression model. The threshold is adjusted iteratively based on the mask ratio, decreasing it when the ratio is too low and increasing it when the specular region is excessively large, with mask dilation applied when necessary. This approach helps keep the specular region from being estimated as too large or too small, supporting accurate detection while reducing damage to the tooth structure.

Following the generation of the initial mask, the masked area may be disproportionately large or small. To address this, a two-stage threshold adjustment process is implemented. The first stage involves a ratio-based static adjustment. If the mask ratio is below a predetermined minimum value (e.g., 10%), then the threshold remains unchanged. Conversely, if the ratio exceeds the maximum value (e.g., 35%), the threshold is capped at the upper limit (e.g., 1.05). For ratios between these values, the threshold is adjusted using a linear interpolation method between the minimum and maximum thresholds. This procedure aims to prevent mask distortion owing to threshold variations by applying normalized corrections in advance, thereby ensuring that the mask area is neither excessively small nor large. The second stage involves a condition-based dynamic adjustment that iteratively modifies the threshold by considering the structural characteristics and distribution of the mask. Figure 6 illustrates the conditional branching flow at this stage.

Initially, if the mask area ratio falls below the predetermined minimum criterion, the threshold is marginally reduced, and the mask is regenerated. Subsequently, if the mask area ratio exceeds the minimum ratio and the threshold has been adjusted at least once, a morphological dilation operation is performed to expand the mask boundaries. This dilation operation can compensate for minor defect areas and incorporate specular highlight boundaries more naturally [4]. Finally, if the area of the largest specular highlight region within the mask exceeds a certain ratio of the entire image, the threshold is increased, and the mask is regenerated. By iteratively applying this process and adjusting the threshold, a sophisticated masking result that reflects the morphological characteristics of the specular highlights and illumination distribution can be achieved. This combined method of static and dynamic adjustments facilitates comprehensive masking in the initial stages of specular highlight detection and subsequently generates a detailed and stable mask by analyzing the mask ratios and connected structures. The finalized mask serves as an accurate and consistent input in the subsequent image inpainting stage, acting as a core element that determines the restoration quality of the entire pipeline.

### 2.6. Image Inpainting

In the final phase, inpainting is employed to restore the masked specular highlight regions, ensuring coherence with the adjacent dental tissue. Inpainting is a technique that visually and naturally reconstructs damaged or occluded image areas by utilizing surrounding information and is extensively applied in the fields of computer vision and medical imaging [4,16]. The essence of this technique lies in achieving a seamless integration of the restored area with the original image by considering the structural and textural context of the surroundings beyond mere color interpolation.

To restore the affected area following the removal of specular highlights from the tooth surface, this study employs an AOT-based generator network [16], a model designed for high-resolution image inpainting. The available pretrained weights provided by Zeng et al. [16] were used directly at inference, without any additional fine-tuning on the dental images of this study, and only the generator component of the model was used to restore the masked regions. The masked image and its corresponding binary mask serve as inputs, enabling the model to analyze the visual context outside the masked region and fill in the masked area. The restored output then undergoes post-processing, in which the generator output is converted to the RGB color space and composited with the original image to produce the final output image, so that the surface texture of the tooth, previously obscured by specular highlights, is naturally reconstructed.

Consequently, this AOT-based inpainting method effectively addresses the structural distortions or visual discrepancies that may arise following the removal of specular highlights. By better preserving the tooth surface texture, the restored images can support subsequent diagnostic and AI analysis processes.

### 2.7. Evaluation Framework: RISC

The principal challenge in assessing the performance of the proposed pipeline lies in the considerable difficulty of obtaining Ground Truth (GT) data owing to the intrinsic characteristics of medical data. Traditional inpainting evaluation metrics, such as the Peak Signal-to-Noise Ratio (PSNR) and Structural Similarity Index (SSIM), rely on direct comparison with the GT [17]. Consequently, in the absence of a GT, these metrics exhibit limitations, such as yielding biased values toward specific outcomes or constraining the evaluation process itself [18,19]. To address these limitations, this study adopted the Re-Inpainting Self-Consistency (RISC) evaluation framework proposed by Chen et al. [19] and adapted it to the assessment of specular highlight removal in intraoral images.

RISC assesses a model’s comprehension of the content it has produced. Inpainting is fundamentally an ill-posed problem in which the content of the missing regions is not uniquely determined, resulting in numerous plausible restoration outcomes, particularly when the mask area is extensive [19]. Conventional GT-based metrics, such as PSNR, introduce issues by skewing numerical values toward a single specific solution among various valid restoration outcomes. In contrast, RISC accommodates multiple valid solutions while simultaneously filtering and regulating only unnatural results that do not align with the context by evaluating the self-consistency of the generated content.

The evaluation process is initiated by generating the first inpainting image, ${\hat{X}}_{1}$, via a specific pipeline ($F_{1}$) under examination. In this study, $F_{1}$ represents the pipeline being evaluated. For comparative analysis, $F_{1}$ was defined separately for two pipelines: the regression-only pipeline, which uses thresholds predicted by the regression model, and the dynamic-adjustment pipeline, which incorporates the proposed Dynamic Adjustment Algorithm after regression-based threshold prediction. Each pipeline was evaluated independently to compare their performance. Subsequently, a second-stage mask, $M_{2}$, is applied to ${\hat{X}}_{1}$. Here, $M_{2}$ is defined by the relationship between a randomly generated patch mask, $M_{p}$, and the initial mask, $M_{1}$, which was used to eliminate specular highlights in the first stage, as follows:

(2) $$M_{2}=1-(1-M_{p})⊙M_{1}$$

The formula employs $M_{1}$ as an initial mask to differentiate between the specular highlight region and the original region, denoted by 0 and 1, respectively. Additionally, $M_{p}$ represents an independent patch mask that is randomly generated for evaluation purposes. This formula enables the selective re-inpainting of only the original areas that remain uncorrupted, as opposed to those already restored by $M_{1}$. This approach assesses the model’s consistency in comprehending and restoring the original context of an image while also mitigating bias arising from style similarity.

The final evaluation score, $D(F_{1})$, for assessing self-consistency is determined by calculating the average similarity between the initial inpainting result, ${\hat{X}}_{1}$, and the K subsequent re-inpainting results [19], as follows:

(3) $$D(F_{1})=\frac{1}{k}\sum_{i=1}^{K}d({\hat{X}}_{1},{\hat{X}}_{2i})$$

In this context, ${\hat{X}}_{2i}$ signifies the outcome of the second inpainting process (re-inpainting) achieved through the i-th iteration. The variable d denotes a sub-metric employed to assess the similarity between two images. This study employs PSNR, SSIM, and LPIPS to validate the reliability of the results from multiple perspectives. First, the PSNR assesses pixel-level accuracy by quantifying the ratio of the maximum possible signal power to the noise power within the image [17]. Second, SSIM evaluates structural similarity, which aligns with human visual perception by incorporating luminance, contrast, and structural information [17]. Finally, LPIPS serves as a metric for perceptual similarity, leveraging the feature extraction capabilities of deep learning models, and is distinguished by its ability to best reflect the visual naturalness perceived by humans, surpassing mere pixel-level differences [18].

The RISC method employed in this study offers distinct advantages that set it apart from the traditional evaluation systems. Primarily, unlike existing methods that require GT, RISC can compute scores without the need for GT. Additionally, it assesses whether the original results are consistently restored during re-inpainting, extending beyond the pixel quality of simple local regions, thereby capturing the global context consistency with greater precision. This capability has been demonstrated to correlate strongly with human visual judgment. Importantly, as it does not rely on distribution distance, it maintains high reliability even in scenarios with limited data or when individual images require independent evaluation. Consequently, RISC addresses the inherent limitations of actual clinical data and serves as a fundamental basis for scientifically and logically ensuring the quality of the pipeline in this study.

### 2.8. Clinical Expert Evaluation

To assess the clinical feasibility of the proposed algorithm, a qualitative evaluation was conducted by four dental experts. The 50 cases used for this evaluation were randomly drawn from the 2463-image evaluation set, and across the four experts, these 50 cases yielded 200 preference responses. For each case, the Original image, the Method A result (regression only), and the Method B result (dynamic threshold adjustment) were presented simultaneously and side by side, allowing direct comparison at a glance. To standardize viewing conditions, the evaluation was conducted through a custom-developed survey platform, and the evaluators were given prior guidance on screen brightness adjustment. All four experts were blinded to which method had produced each image. Although the three images were labeled by their position, the evaluators remained blinded as to which of Method A and Method B corresponded to the regression-only and the dynamic adjustment methods. For each preference judgment, the evaluators were explicitly instructed to consider whether the specular highlights had been effectively removed and whether the anatomical structure of the teeth had been well restored. The left-to-right arrangement of the three images, namely the Original on the left, Method A in the middle, and Method B on the right, was fixed across all 50 cases and was not counterbalanced on a case-by-case basis. The influence of this fixed arrangement is expected to be limited, because the evaluators remained blinded as to which position corresponded to the regression-only or the dynamic adjustment method, so that any positional preference could not map systematically onto a specific algorithm.

The evaluation was structured into two segments. In the first segment, the comparative preference between the regression-only method (Method A) and the proposed adaptive algorithm (Method B) was assessed. The collected responses were analyzed using the Fleiss’ Kappa index [20] to assess inter-rater reliability and the level of agreement among the four evaluators. In addition, a Wilcoxon Signed-Rank Test [21], a nonparametric statistical hypothesis test, was performed to determine whether the observed difference in preference between the two methods was statistically significant. To account for the clustered structure of the data, this test was conducted at the case level. The four raters’ scores for each case were averaged to yield 50 case-level preference scores, which were then tested against zero. The matched rank-biserial correlation was reported as the effect size, and 95% confidence intervals for the preference proportions were obtained using the Wilson method.

In Section 2, a comprehensive survey employing a Likert scale was conducted to evaluate specific criteria, including tooth surface texture preservation, clinical utility, and significance of data standardization. Given the ordinal nature of the Likert scale [22], the median was calculated to mitigate statistical distortion caused by extreme response values and accurately determine the overall central tendency of the responses. Furthermore, to assess the dispersion of responses around the median, the Interquartile Range (IQR) was calculated, thereby enabling an objective analysis.

## 3. Results

This study developed an automated pipeline that integrates a regression model with a dynamic adjustment algorithm to manage specular highlights in intraoral clinical images. This section evaluates, through both visual and quantitative analyses, the pipeline’s ability to autonomously identify optimal specular highlight regions and perform restoration while preserving the tooth surface texture.

**Figure 7:**
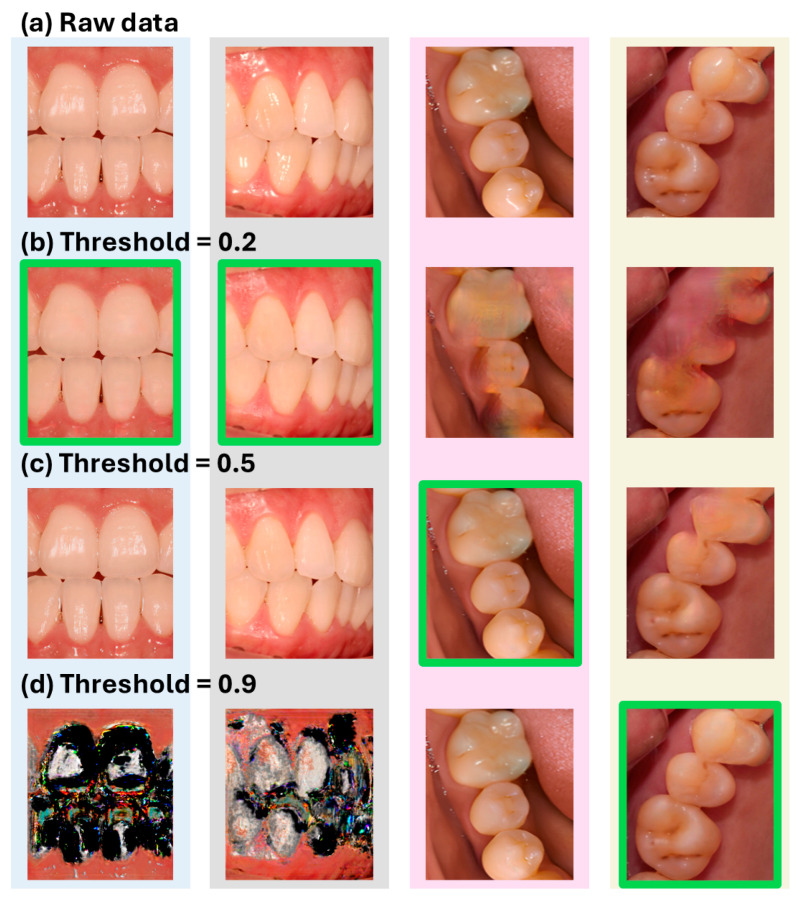
**Comparison of specular highlight removal results according to threshold variation.** The results provide visual evidence of the limitation of applying a fixed threshold across different images. (**b**–**d**), Specular highlight removal results obtained by applying fixed thresholds of 0.2 (**b**), 0.5 (**c**), and 0.9 (**d**), respectively. Each column shows the results obtained by processing the same input image shown in (**a**) under different threshold conditions, enabling direct comparison of threshold-dependent restoration outcomes. The green boxes mark the most naturally restored result for each image. Notably, the optimal threshold varies significantly by image: 0.2 for the first two columns, 0.5 for the third, and 0.9 for the fourth. This confirms that a fixed threshold approach is ineffective and that an image-specific adaptive method is necessary.

Intraoral clinical images obtained in dental environments display significant variability in the intensity of specular highlights, which is contingent on the illumination intensity and imaging angle [5,10]. When a uniform threshold is applied across all images, some images do not have specular highlights adequately removed, which impedes interpretation. Conversely, excessive setting of the specular highlight regions can result in the loss of detailed structural information from the tooth enamel. These limitations are illustrated in Figure 7. An analysis of four representative cases with varying thresholds (T=0.2, 0.5, 0.9) confirmed that a uniform threshold yields disparate results. The first and second cases exhibited the most natural removal performance at T=0.2, whereas at T=0.9, the structural integrity of the teeth was compromised. In contrast, the third case demonstrated optimal results at T=0.5, and the fourth case at T=0.9, with other thresholds leading to issues such as blurred tooth boundaries or residual specular highlights. This underscores the necessity of an algorithm that autonomously determines the optimal threshold for each image while minimizing user intervention [4,6].

To quantitatively demonstrate this variability, the RISC metrics were assessed in relation to alterations in the fixed thresholds, as presented in Table 2. The experimental findings indicate that as the threshold increases from 0.2 to 0.9, the LPIPS value increases from 0.036 to 0.065, whereas the PSNR and SSIM values decrease substantially from 40.9 to 28.5 and from 0.97 to 0.90, respectively. This observation suggests that restoration consistency can be substantially compromised depending on the threshold setting, thereby underscoring the necessity of an individualized optimization algorithm capable of adapting to diverse patient cases.

**Figure 8:**
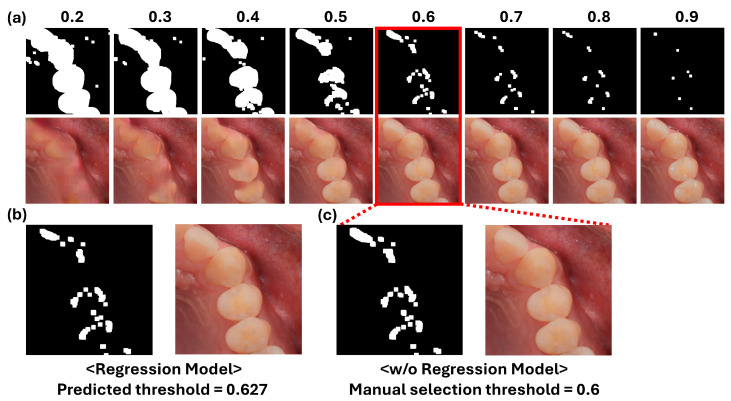
**Validation of regression model for predicting optimal threshold.** The comparison demonstrates that the regression model predicts thresholds consistent with human visual perception. (**a**) Masks generated by thresholds ranging from 0.2 to 0.9, showing the reduction in masked area as the threshold increases. (**b**) Result obtained using the threshold predicted by the regression model (0.627). (**c**) Result obtained using the manually selected optimal threshold (0.6) without using the regression model. The numerical proximity between the predicted and manually selected thresholds, together with the visual similarity of the resulting masks and restored images, confirms the model’s high prediction accuracy.

The automation performance of the regression model, which was introduced to resolve the inconvenience of manually searching for the optimal threshold for each image, is demonstrated in Figure 8. As shown in Figure 8a, a trade-off exists, where a lower threshold results in excessive masking that damages the tooth structure, whereas a higher threshold leads to insufficient specular highlight removal. The threshold predicted by the regression model (0.627, Figure 8b) showed high similarity in both numerical value and visual results to the optimal value manually selected by a clinician through visual inspection (0.6, Figure 8c). This indicates that the algorithm has high reliability in performing standardized preprocessing without subjective expert intervention.

**Figure 9:**
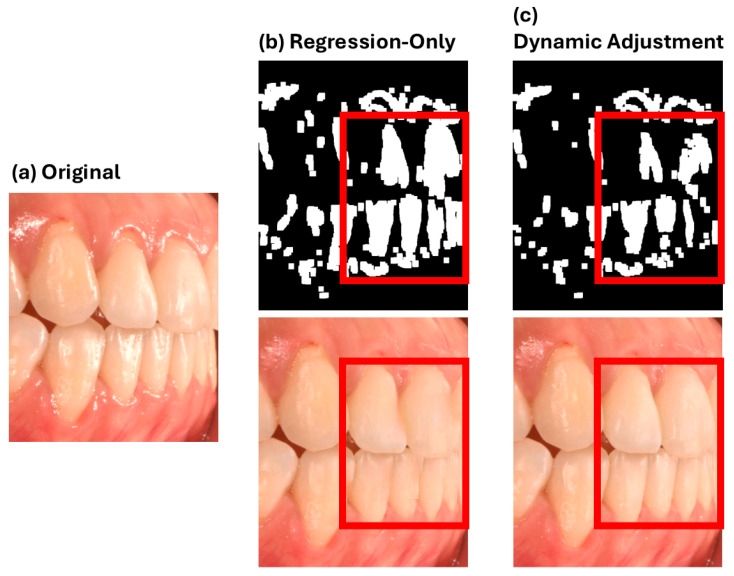
**Comparison of specular highlight removal results with and without dynamic adjustment algorithm.** The comparison highlights the necessity of the dynamic threshold adjustment algorithm for precise restoration. (**a**) Original image. (**b**) Results obtained without the adjustment (regression only) show that the predicted threshold can sometimes define the specular region too broadly, as indicated by the red boxes, causing damage to the tooth structure. (**c**) Results with the dynamic threshold adjustment demonstrate that the algorithm effectively corrects these deviations by preventing both excessive and insufficient masking, thereby supporting more stable and accurate specular highlight removal.

Although the regression model initially yielded promising predictions, it was insufficient to address all cases independently owing to the inherent complexity of the clinical data. Figure 9 demonstrates the essential role of the dynamic adjustment algorithm in addressing these issues. When the regression model was employed alone, the masking area exceeded the actual specular highlights, resulting in damage to the intrinsic anatomical structure of the teeth (Figure 9b). Conversely, after implementing the dynamic adjustment, the mask boundaries were precisely calibrated, enabling the selective removal of specular highlights while reducing structural distortion (Figure 9c). This finding suggests that the proposed pipeline functions as an adaptive system capable of responding to the unique characteristics of individual images beyond static prediction.

**Figure 10:**
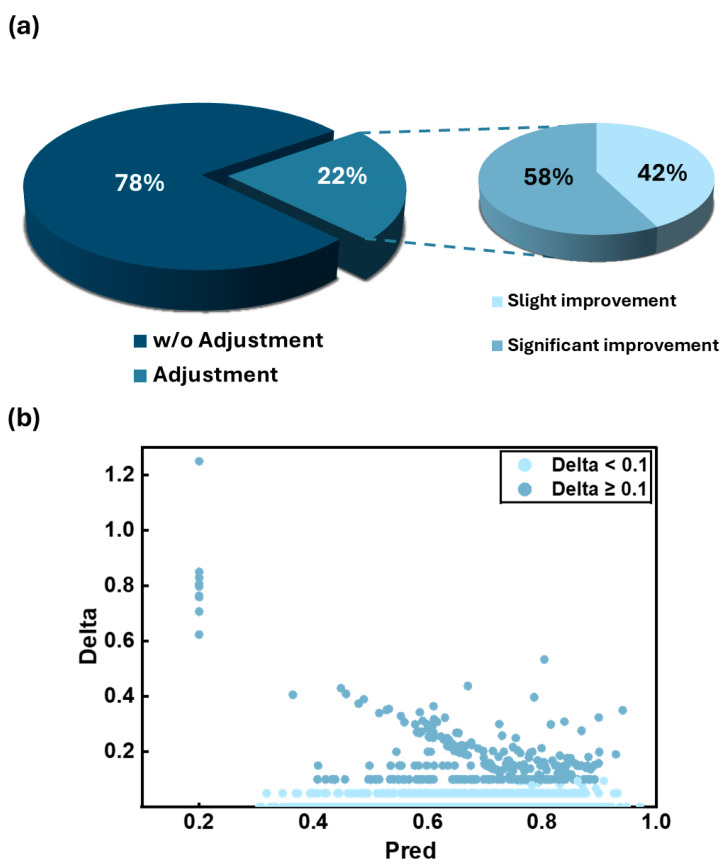
**Quantitative evidence of dynamic threshold adjustment necessity.** Statistical analysis verifying the essential role of the dynamic adjustment algorithm in the pipeline. (**a**) The charts reveal that 22% of the total dataset required dynamic adjustment, indicating that the regression model alone is insufficient for approximately one-fifth of the cases. Among these adjusted cases, 58% showed significant improvement (Δ≥0.1) and 42% showed minor improvement (Δ<0.1), proving that the algorithm consistently enhances specular removal performance. (**b**) The scatter plot displays the distribution of the threshold adjustments (Δ) relative to the predicted thresholds. The wide distribution of Δ across the *x*-axis demonstrates that discrepancies between the predicted and optimal thresholds are not limited to a specific range but occur diversely. This confirms that the dynamic adjustment algorithm functions as a robust safeguard, effectively correcting unpredictable deviations across the entire spectrum of predictions that the regression model cannot inherently capture.

The quantitative impact of the proposed adaptive calibration mechanism on a large-scale dataset is shown in Figure 10. Of the 2463 data points, the dynamic adjustment algorithm intervened to calibrate the threshold in approximately 22% of the cases (551 images) (Figure 10a). Among these calibrated instances, 58% exhibited a noticeable improvement in quality discernible to the naked eye (Delta≥0.1), whereas 42% underwent a fine calibration process to enhance data precision (Delta<0.1). The scatter plot in Figure 10b depicts the difference (*Delta*) between the initial predicted value of the regression model and the final adjusted value, indicating that the algorithm plays an important role in preserving the tooth surface texture by compensating for the limitations of the regression model.

**Figure 11:**
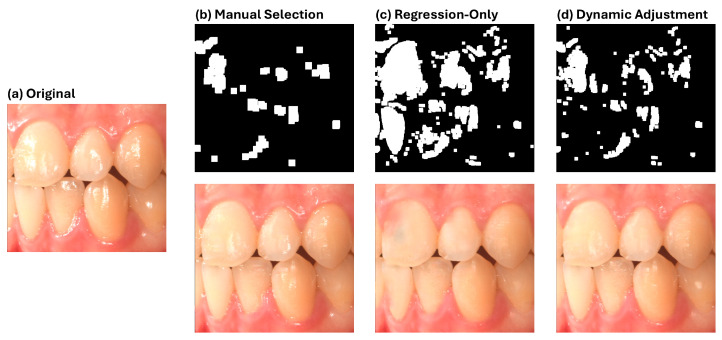
**Comparison of optimal threshold, regression-only, and dynamic adjustment approaches.** The comparison evaluates the proposed dynamic adjustment method against regression-based and manual approaches. (**a**) Original input image. (**b**) Result using the manually selected optimal threshold, showing substantial residual specular highlights due to under-segmentation. (**c**) Result using the regression model’s predicted threshold, showing visible damage to the tooth structure due to over-segmentation of the mask. (**d**) Result using the dynamic threshold adjustment algorithm. The dynamic adjustment method achieves superior performance by balancing the threshold between (**b**,**c**), effectively removing highlights while preserving the tooth structure.

Figure 11 presents a comprehensive comparison of manual selection, regression-only, and the proposed method incorporating dynamic adjustment. For the same image, manual selection (Figure 11b) demonstrated limitations in adequately capturing specular highlights, whereas the regression-only method (Figure 11c) exhibited excessive masking issues that compromised the tooth structure quality. In contrast, the proposed adaptive pipeline (Figure 11d) most meticulously identified the specular highlight regions among the three methods and consistently removed visual noise while fully preserving the tooth surface texture. This result indicates that the automated adaptive approach is more sophisticated than conventional methods employing fixed thresholds and can secure standardized high-fidelity intraoral clinical image data, surpassing the mere enhancement of user convenience.

The qualitative superiority is reaffirmed through the RISC-based quantitative comparison in Table 3. The analysis results indicate that as the mask area ratio and patch size decrease, conditions more conducive to model handling are established, resulting in a tendency for performance enhancement across all metrics, including LPIPS, PSNR, and SSIM. The dynamic adjustment algorithm maximizes self-consistency by adaptively optimizing the mask size and reducing the restoration uncertainty. This ultimately demonstrates that more sophisticated and standardized high-fidelity intraoral clinical images can be obtained compared with conventional methods employing fixed thresholds.

In a survey in which four dental experts independently assessed the same 50 cases, yielding 200 preference responses in total, the proposed algorithm (Method B) received a higher clinical preference than the regression-only method (Method A). Method B was preferred in 69.5% of responses (139 instances, 95% CI 62.8 to 75.5), Method A in 11.0% (22 instances, 95% CI 7.4 to 16.1), and 19.5% (39 instances, 95% CI 14.6 to 25.5) indicated no clear visual difference. To account for the clustered structure of the responses, the preference was tested at the case level rather than by treating the 200 responses as independent. The four raters’ scores for each case were averaged, and the resulting 50 case-level scores were compared against zero using the Wilcoxon Signed-Rank Test, which was significant (Z=−5.41,  p<0.001) with a large effect size (r = 0.825). A complementary sign test was consistent, with Method B preferred over Method A in 39 of the 50 cases (*p* < 0.001). The Fleiss’ Kappa index, which measures agreement among multiple evaluators, was 0.365, corresponding to a Fair Agreement level [23].

In the qualitative assessment of items using a 5-point Likert scale, the proposed method achieved commendable scores. Specifically, for item 3.2, which inquired whether the automated preprocessing would facilitate the acquisition of large-scale, high-fidelity datasets for future advanced diagnostic AI training, all experts demonstrated consistent support, approaching a perfect score with a median of 5.0 (IQR 4.8 to 5.0). Furthermore, for item 1.2, which addressed whether the proposed technology mitigated the issue of excessive masking that results in the loss of normal dental tissue information, a median of 4.0 (IQR 3.8 to 4.0) was achieved, and for item 2, which addressed whether clear data provides a practical foundation for clinician interpretation and long-term follow-up, a median of 4.0 (IQR 4.0 to 4.2) was obtained.

## 4. Discussion

In this study, an automated pipeline was proposed to reduce specular highlights in intraoral images by integrating regression-based threshold prediction with a condition-based dynamic adjustment algorithm. This method showed improved stability and consistency in removing specular highlights compared with conventional fixed threshold approaches, addressing several of their limitations.

Table 2 presents an analysis of the quantitative metrics under fixed threshold conditions. A reduction in the threshold correlates with enhanced inpainting performance across all metrics, namely LPIPS, PSNR, and SSIM. This phenomenon is intrinsically linked to the capacity of the RISC evaluation framework to assess the model’s consistency in comprehending and restoring images. When a high threshold (T=0.9) is applied, pronounced specular highlight patterns persist within the image, presenting highly unnatural and irregular patterns that challenge the model’s predictive and reproductive capabilities. During the RISC re-inpainting process, when the corresponding region is masked again, the model endeavors to generate clean dental tissue based on surrounding information rather than restoring the specular highlights. This results in an increased visual discrepancy between the residual specular highlights and the newly generated tissue, leading to an increase in the LPIPS and a decrease in the PSNR and SSIM. Conversely, when specular highlights are largely removed through a low threshold, the image is dominated by stable dental textures that the model can readily manage, which leads to higher self-consistency during the re-inpainting process.

As shown in Table 3, the inpainting performance metrics tend to improve as the mask ratio and patch size decrease. This occurs because a smaller mask ratio leaves more surrounding context for the model to reference, and a smaller patch size shortens the distance between the occluded region and that context, allowing restoration to proceed largely by interpolation from adjacent pixels rather than by generating new structures [16,19]. The dynamic adjustment algorithm was designed with these characteristics in mind. By analyzing the area and distribution of the mask and adjusting the threshold accordingly, it prevents the regression model from overpredicting the threshold, which could otherwise leave residual specular highlights or produce an excessively large mask, and thereby keeps the mask in a range that the inpainting model can handle reliably.

The metrics in Table 3, however, should be interpreted with care. The regression-only method yields marginally higher RISC values than the proposed dynamic adjustment method, but this small advantage likely reflects a limitation of the inpainting evaluation metrics rather than better restoration. When complex textures such as the curvatures of tooth surfaces are over-removed and flattened, predicting and restoring the simplified result becomes easier for the inpainting model, which can inflate PSNR, SSIM, and LPIPS scores [17,18]. A slight numerical advantage on these metrics therefore does not necessarily indicate better restoration and may instead signal a loss of fine surface detail. This interpretation is the central point of the following analyses, which provide qualitative and distributional evidence for it.

**Figure 12:**
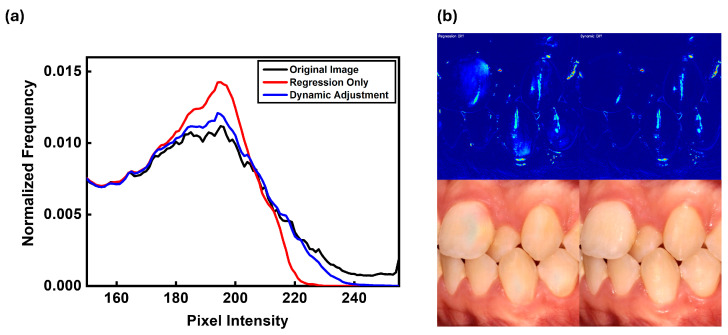
Qualitative comparison of specular highlight removal and restoration performance using pixel intensity distribution and difference maps. (**a**) Comparison of pixel intensity distribution between the original image and each model: The Dynamic Adjustment method preserves the original distribution profile while gradually correcting the high-intensity regions. (**b**) Difference maps (**top**) and final inpainting results (**bottom**) for each method: The results demonstrate that the Dynamic Adjustment approach achieves natural restoration without losing tooth texture relative to the regression-only model.

Figure 12 illustrates this point qualitatively. The upper difference map in Figure 12b shows that the regression-only method marks a broader region as a specular highlight than the dynamic adjustment method, yet the corresponding restored images reveal that this broader masking removes part of the intrinsic tooth texture. The dynamic adjustment method, by contrast, follows the surrounding texture more closely and yields a more natural restoration. The pixel intensity distribution in Figure 12a is consistent with this observation, as the distribution produced by the dynamic adjustment algorithm remains closer to that of the original image. In the high-intensity region (220 to 255) where specular highlights are concentrated, the frequency drops sharply and detail is lost when only the regression model is used, whereas the dynamic adjustment produces a gentler curve that reflects a more gradual removal of the highlights.

To complement the qualitative findings described above and to cross-check the limitations of the RISC evaluation metrics, additional quantitative measures were introduced. Specifically, the Wasserstein distance (WD) [24] was used to quantify the difference in shape between the pixel intensity distributions, and the Kullback–Leibler divergence (KLD) [25] was used to evaluate the extent of information loss. The proposed dynamic adjustment method achieved a WD value of 1.3505, reducing the distribution error by more than half relative to the method employing only the regression model (2.9601). The KLD likewise decreased from 0.3451 to 0.0618, indicating a higher degree of preservation of the original pixel distribution. These results suggest that the marginally higher RISC scores previously observed for the regression-only method can be attributed to a loss of surface texture information. This in turn indicates that the method incorporating the dynamic adjustment algorithm achieves stable restoration while better preserving the surface texture. The proposed algorithm therefore offers a more balanced tradeoff between restoration consistency, as measured by RISC, and the preservation of surface texture, as measured by WD and KLD, supporting its usefulness for retaining the fine surface detail relevant to clinical interpretation.

**Figure 13:**
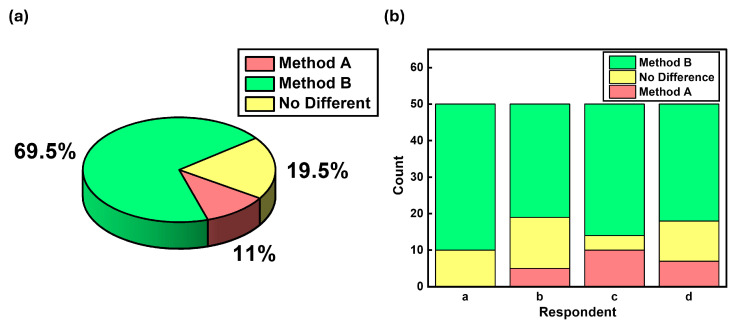
**Clinical expert evaluation of the comparative preference between the regression-only method (Method A) and the proposed dynamic adjustment method (Method B).** (**a**) Overall distribution of 200 evaluation responses from four dental experts across 50 randomized cases. The proposed dynamic adjustment method (Method B) was preferred in 69.5% of the responses, whereas the regression-only method (Method A) was preferred in 11.0%, and no clear difference was reported in 19.5%. (**b**) Response distribution for each evaluator, showing a consistent tendency among all experts to select Method B more frequently.

The clinical expert evaluation provides supporting evidence for the practical utility of the proposed dynamic adjustment algorithm. As shown in Table 4 and the pie chart in Figure 13, the proposed algorithm (Method B) received a higher preference than the regression-only method (Method A), accounting for a preference share of 69.5%. The response distribution among the four individual evaluators also showed a consistent tendency for all clinicians to select Method B most frequently, and this preference was statistically significant according to the Wilcoxon Signed-Rank Test reported in Table 4. The Fleiss’ Kappa index was 0.365, corresponding to a Fair Agreement level [23], which should be interpreted with caution. This level of agreement likely suggests subtle differences in each clinician’s subjective criteria for judging detail preservation and masking intensity rather than a lack of overall consensus on the preferred method. Even so, the preference for Method B was consistent across all four evaluators individually (Figure 13b), indicating that the overall preference converged on the proposed algorithm despite the limited agreement level. Because the 200 preference responses were clustered by both evaluator and case, the four raters’ scores within each case were averaged so that the analysis was based on 50 independent case-level observations rather than 200 clustered responses.

The survey results presented in Table 5 provide further insight into how the proposed method was perceived by clinical experts. For the item assessing whether Method B addresses over-masking, which may lead to the loss of information from normal dental tissue, the responses yielded a median score of 4.0 with a narrow IQR of 3.8 to 4.0, suggesting that the experts generally regarded the method as reducing excessive masking while preserving fine surface texture. Similarly, the item evaluating whether clear image data provide practical evidence for clinical interpretation and follow-up received a median score of 4.0 with an IQR of 4.0 to 4.2, indicating consistently positive expert responses. The automated pipeline was also considered effective in reducing user-dependent bias arising from manual masking and supporting objective data reliability [26], as reflected by a median score of 4.0 and an IQR of 4.0 to 4.0. Among the survey items, the most favorably rated was the perceived contribution to securing large-scale datasets for future training of diagnostic AI models, which received a median score of 5.0 and an IQR of 4.8 to 5.0. These responses indicate that the experts viewed the method as a useful means of supporting more consistent dental image data. Taken together, the alignment between the quantitative analysis and the expert responses suggests the potential applicability of the proposed algorithm as a preprocessing tool for dental image standardization and medical AI data preparation [27].

The proposed preprocessing may also offer benefits for downstream diagnostic AI models. By reducing the noise introduced by specular highlights while preserving the surface texture of the enamel, the pipeline can help provide cleaner and more consistent input for model training. Tasks that could plausibly benefit include lesion detection models that are sensitive to subtle surface changes such as enamel microcracks or early caries, and longitudinal monitoring models that compare the same site across repeated examinations. As this study focused on the specular highlight removal pipeline itself, it did not extend to training or evaluating a downstream diagnostic model. Quantitatively validating these potential benefits on actual diagnostic tasks is planned as part of future research.

At the same time, the way generative inpainting operates introduces a risk that warrants particular attention. Models such as AOT GAN restore a masked region by statistically inferring plausible content from the surrounding texture rather than by recovering the true underlying surface. As a result, in some cases the model may introduce microstructures that were not actually present, such as spurious enamel cracks, or it may smooth over subtle features that should have been retained. Such cases are expected to be infrequent, given the small and localized masked regions typical of specular highlights, but the possibility cannot be entirely excluded. For this reason, it is preferable to regard the proposed pipeline as a preprocessing aid that standardizes image quality and to be cautious about relying on it directly for diagnostic decisions. The restored images are intended to support clinical interpretation and downstream analysis rather than to replace the judgment of clinicians or appropriately validated diagnostic systems.

Although the present validation relied on synthetic images, the characteristics of this dataset merit further comment. According to the documentation provided by AI Hub, the synthetic images were generated from the intraoral photographs of 7000 real dental hospital patients and were designed to reflect the imaging angles and clinical conditions of diverse real cases. While this does not amount to validation on actual clinical photographs, it indicates that the dataset retains a meaningful degree of clinical realism derived from real patient data. The synthetic setting can therefore be viewed as a controlled yet clinically grounded environment that is suitable for an initial proof-of-concept evaluation, with validation on real clinical photographs remaining a key task to be addressed.

The evaluation strategy adopted in this study also differs from that of prior work, which bears on how the present results should be compared. Earlier specular highlight removal studies, including deep learning-based methods developed for natural images [3,10], have generally assessed restoration performance using ground truth-based metrics such as PSNR and SSIM. Such metrics are informative for quantifying pixel-level accuracy or structural similarity when a reference image with the specular highlights already removed is available. For the dental intraoral images addressed in this study, however, a ground truth image of this kind is inherently difficult to obtain. The RISC framework was therefore employed to evaluate the self-consistency of the restoration without requiring ground truth, and Wasserstein distance and KL divergence were further used to examine information loss at the level of the pixel distribution. Given that these approaches differ from prior work in their underlying premise, in the availability of ground truth, and in the type of images involved, namely natural or endoscopic images as opposed to dental intraoral images, the RISC-based LPIPS, PSNR, and SSIM values, together with the Wasserstein and KL divergence values reported here, are not readily comparable with those reported in earlier studies on a common basis. A side-by-side quantitative comparison of existing methods and the proposed pipeline with an identical dataset and evaluation protocol was likewise difficult in the present study, given the limited availability of a suitable publicly accessible baseline.

Several limitations of this study should be acknowledged. First, the validation was based on a synthetic dataset, and validation on real clinical photographs involving dental prostheses, metallic restorations, orthodontic appliances, saliva, and focus variation was not performed. The present results therefore demonstrate performance under controlled conditions rather than across the full range of clinically relevant intraoral images. Second, the threshold labels used to train the regression model were judged independently by five raters, without calibration, consensus procedures, or formal inter-rater reliability assessment, so the subjectivity of these reference labels was not quantified. Third, as discussed above, a direct quantitative comparison with existing specular highlight removal techniques or other deep learning-based restoration methods was limited by the lack of a suitable publicly accessible baseline for dental intraoral images. Fourth, the quantitative metrics used in this study, namely PSNR, SSIM, LPIPS, Wasserstein distance, and KL divergence, assess visual consistency and distributional similarity but do not directly demonstrate the preservation of diagnostically relevant microstructures such as enamel microcracks or early caries. Fifth, generative inpainting models such as AOT GAN carry an inherent risk of hallucination, in that they may plausibly generate structures that do not actually exist or, conversely, smooth over genuine lesions. To address these limitations, future work will sequentially pursue prospective validation on real clinical photographs, a labeling protocol involving multiple independent and blinded raters with standardized calibration, quantitative comparisons with classical and deep learning-based restoration methods, and task-specific validation based on microstructures labeled by clinical experts.

Taken together, these findings indicate that the proposed algorithm reduces specular highlights while better preserving the fine surface texture of the teeth than the baseline approaches. By integrating the dynamic adjustment algorithm, the method mitigates the over-masking that can occur when the regression model is used alone, thereby retaining surface detail that would otherwise be lost. These results, however, were obtained under controlled conditions using synthetic images, so claims of clinical applicability should be made with caution. Even so, within these limitations, the proposed algorithm is meaningful in that it can serve as a basis for standardized preprocessing in digital dentistry and medical AI data preparation.

## 5. Conclusions

This study introduces an automated pipeline that integrates regression-based threshold prediction with a structure-adaptive dynamic adjustment algorithm to reduce specular highlights, which can impede the diagnostic interpretation of intraoral clinical images [5,6,28]. Based on statistical data from the HSV color space, the optimal threshold for each image was automatically determined, and precise specular highlight regions were extracted through an adaptive calibration process that accounted for the mask area ratios and their distributions. Subsequently, an AOT-based inpainting technique was employed to naturally restore the removed areas, ensuring coherence with the surrounding dental tissue.

Experimental results utilizing the RISC framework [19] suggest that the proposed method may reduce the performance variance and boundary distortion commonly associated with traditional fixed-threshold approaches [4,6]. Importantly, both quantitative and qualitative analyses indicate that the quality of inpainting transcends mere numerical consistency, emphasizing the preservation of the tooth surface texture [17,18]. A key contribution of this study is the balance between quantitative consistency and qualitative realism. The dynamic adjustment algorithm helps mitigate the risk of information loss due to over-masking, which is a prevalent issue when regression models are used in isolation. Moreover, the application of the RISC evaluation method illustrates that objective quality assessment is achievable even in clinical settings where Ground Truth data are challenging to obtain [19,27,29], which holds considerable academic significance.

As a proof-of-concept evaluation conducted on synthetic intraoral images, the proposed pipeline applies uniform statistical criteria in place of manual preprocessing, an approach intended to reduce the observer bias inherent in manual masking [26], while better preserving tooth surface texture than the baseline approaches. These results suggest that the method may, in the future, provide clinicians with more consistent visual data for long-term follow-up [1] and contribute to the standardization of large-scale dental image datasets for training diagnostic AI models [27]. However, these broader applications were not directly tested in this study and require validation on real clinical photographs. Accordingly, future research will focus on prospective validation using real clinical photographs, including cases with dental prostheses, metallic restorations, orthodontic appliances, saliva, and focus variation, and on extending the model to incorporate multiple characteristics of specular highlights beyond a single threshold.

## Figures and Tables

**Table 1:** Composition of the image datasets used in this study.

Image Stage/Set	Images	Purpose
**Regression development**	14,514	Development of the HSV threshold regression model
Training set	452	Manually selected optimal thresholds used as ground truth
Threshold prediction set	14,062	Generalization check only, not used in final results
**Pipeline evaluation**	2463	Application and evaluation of the full three-stage pipeline
Expert evaluation set	50	Clinical expert preference survey

**Table 2:** Quantitative comparison of RISC metrics under fixed threshold values of 0.2, 0.5, 0.7, and 0.9.

	Fixed Threshold			
	Threshold = 0.2	Threshold = 0.5	Threshold = 0.7	Threshold = 0.9
LPIPS	0.03631	0.05651	0.06336	0.06508
PSNR	40.90854	32.25258	29.50434	28.54358
SSIM	0.97038	0.92101	0.90485	0.90072

**Table 3:** RISC-based quantitative comparison between regression-only processing and dynamic adjustment under different mask ratios and patch sizes.

	Regression Only				Dynamic Adjustment			
	Mask Ratio = 0.4		Mask Ratio = 0.2		Mask Ratio = 0.4		Mask Ratio = 0.2	
	Patch Size = 32	Patch Size = 16	Patch Size = 32	Patch Size = 16	Patch Size = 32	Patch Size = 16	Patch Size = 32	Patch Size = 16
LPIPS	0.07867	0.06633	0.05303	0.03491	0.08186	0.06824	0.05384	0.03529
PSNR	35.26916	37.64480	38.69754	40.85675	34.90796	37.27955	38.35015	40.53178
SSIM	0.94996	0.95105	0.96719	0.97120	0.94852	0.95029	0.96657	0.97093

**Table 4:** Statistical analysis of preference and inter-rater reliability (N = 50 cases, four evaluators).

Analysis Category	Measurement/Method	Value	Interpretation
Inter-rater Reliability	Fleiss’ Kappa	0.365	Fair Agreement
Comparative Preference	Method A Frequency	22 (11.0%, 95% CI 7.4–16.1)	-
	Method B Frequency	139 (69.5%, 95% CI 62.8–75.5)	-
	No Difference	39 (19.5%, 95% CI 14.6–25.5)	-
Statistical Significance	Wilcoxon Signed-Rank Test	$Z^{*}=-5.41,p<0.001,$ r=0.825	Significant Difference
Sensitivity Check	Sign Test (B vs. A, per case)	39 vs. 4, p<0.001	Consistent with Wilcoxon

* Z-score approximation for the Wilcoxon signed-rank test; r = matched rank-biserial correlation; confidence intervals computed using the Wilson method.

**Table 5:** Clinical expert evaluation of tooth surface texture preservation, clinical utility, and data standardization using a 5-point Likert scale.

Item	Question	Median (IQR, Q1–Q3)
1.1	Method B preserves anatomical details, including the characteristic texture and fine surface curvature of enamel, without distortion during specular highlight removal.	3.5 (3.0–4.2)
1.2	Method B effectively addresses over-masking, which may result in the loss of information from normal dental tissue.	4.0 (3.8–4.0)
2.	Clear image data that are not distorted by specular highlights provide practical evidence for accurate clinical interpretation and long-term follow-up of patients.	4.0 (4.0–4.2)
3.1	The automated pipeline of Method B eliminates user-dependent bias associated with manual masking and ensures objective data reliability.	4.0 (4.0–4.0)
3.2	The automated preprocessing procedure contributes to the acquisition of large-scale, high-fidelity datasets for future training of advanced diagnostic AI models.	5.0 (4.8–5.0)

## Data Availability

The source dataset used in this study is publicly available from AI Hub, provided by the National Information Society Agency, Republic of Korea, under the dataset “Synthetic Oral Image Data” (dataset no. 71688), subject to the AI Hub terms of use and access requirements. Available online: https://aihub.or.kr/aihubdata/data/view.do?aihubDataSe=data&dataSetSn=71688 (accessed on 21 October 2025). The processed data, analysis results, and the code used for regression model training, threshold optimization, and pipeline integration are available from the corresponding author upon reasonable request.

## Abbreviations

The following abbreviations are used in this manuscript:

HSV	Hue, Saturation, Value
RGB	Red, Green, Blue
LAB	$L^{*},\;$$a^{*},\;$$b^{*}$
AOT-GAN	Aggregated Contextual Transformation Generative Adversarial Networks
RISC	Re-Inpainting Self-Consistency
GT	Ground Truth
PSNR	Peak Signal-to-Noise Ratio
SSIM	Structural Similarity Index
LPIPS	Learned Perceptual Image Patch Similarity
IQR	Interquartile Range
KLD	Kullback–Leibler Divergence
